# Effective Connectivity Analysis of Brain Activated Regions during Distracted Driving

**DOI:** 10.3390/brainsci11060690

**Published:** 2021-05-24

**Authors:** Mi-Hyun Choi, Jin-Ju Jung, Je-Hyeop Lee, Ye-Jin Kim, Kyu-Beom Kim, Hyung-Sik Kim, Jeong-Han Yi, Soon-Cheol Chung

**Affiliations:** Department of Biomedical Engineering, BK21+ Research Institute of Biomedical Engineering, School of ICT Convergence Engineering, College of Science & Technology, Konkuk University, Chungju 27478, Korea; mhchoi0311@gmail.com (M.-H.C.); wjd3621@kku.ac.kr (J.-J.J.); charlie63@kku.ac.kr (J.-H.L.); re5spite@kku.ac.kr (Y.-J.K.); rlarbqja0507@gmail.com (K.-B.K.); hskim98@kku.ac.kr (H.-S.K.); jeong2yi@kku.ac.kr (J.-H.Y.)

**Keywords:** effective connectivity, driving, secondary task (addition task), motor control pathway, motor network of declarative memory

## Abstract

This study aims to use functional magnetic resonance imaging (fMRI) to assess the effective connectivity between the regions of the brain activated when driving and performing a secondary task (addition task). The subjects used an MR-compatible driving simulator ㅊ to manipulate the driving wheel with both hands and control the pedals (accelerator and brake) with their right foot as if they were driving in an actual environment. Effective connectivity analysis was performed for three regions of the right and the left hemispheres with the highest z-scores, and six of the regions of the entire brain (right and left hemisphere) activated during driving by dynamic causal modeling (DCM). In the right hemisphere, a motor control pathway related to movement control for driving performance was discovered; in the left hemisphere, the pathways in the regions related to movement control for driving performance, starting with the region associated with the secondary task, were discovered. In the whole brain, connectivity was discovered in each of the right and left hemispheres. The motor network of declarative memory, which is the connectivity of the right thalamus, left lingual gyrus, and right precentral gyrus, was worth noting. These results seem meaningful, as they demonstrate the connectivity associated with the control of voluntary movement related to memory from human experience, although limited to driving tasks.

## 1. Introduction

The development of functional magnetic resonance imaging (fMRI) technology has made it possible to study the functions and connectivity of brain regions. Research on driving, which requires complex cognitive processing, including attention, learning, memory, and decision making, using a driving simulator is ongoing [1,2,3,4,5]. Michon [6] reported that driving requires complex cognitive processing involving three interacting hierarchical levels: the strategic level (trip planning and route finding), the tactical level (planning of relevant actions based on the current driving context), and the operational level (action execution and perception). The driver must perform the driving task properly while being careful not to make a mistake, and this requires complex cognitive processing. The cognitive-judging process is very important in driving since most traffic accidents are caused by errors in the drivers’ cognition and judgment. More than 90% of these required cognitive and judgment processes are based on the information acquired by vision. For this reason, research on the neurological aspects of driving performance mainly focuses on complex cognition, including vision.

Drivers are often engaged in secondary tasks (radio tuning, dialing a cell phone, eating, or carrying on a conversation). In neuroimaging studies, only research results on neural activation during driving and secondary tasks, such as conversation, auditory language comprehension task, and visual event detection, have been reported [2,4,5,7,8,9].

Cognitive processing is related to the local function as well as the connectivity of regions of the brain. For this reason, studies have recently been conducted to observe connectivity between regions of the brain for various cognitive tasks [10,11,12,13,14,15,16,17]. However, there are limited research reports on brain connectivity for tasks such as driving that require complex cognitive processing. There have been research findings on functional connectivity related to driving performance according to driving experience [10,11]. It was reported that experienced drivers have better capacities for sensation, decision-making, and situational awareness while driving in dangerous conditions in comparison to novice drivers. Between the novice and experienced drivers’ groups, differences in activation and connectivity were observed in regions associated with visual attention, decision-making, and executive control processes: intraparietal sulcus (IPS) [13], frontal eye field (FEF) [14,15], and anterior cingulate cortex (ACC) [13,16]. Recent studies have reported comparative analysis results between vice drivers and experienced drivers (accident-free for 10 years or longer), in which they observed areas of brain activation while performing hazard perception tasks and measuring changes in functional connectivity of visual attention and saliency networks [17]. As a result, both groups showed increased blood oxygen level-dependent (BOLD) activation in occipital, parietal, and frontal areas as they performed hazard perception tasks while driving. Experienced drivers indicated the increased activation in executive attention regions and higher functional connectivity between bilateral occipital cortices and salience network when performing hazard perception tasks [17]. Another preceding study carried out a simulated driving and task-cueing experiment simultaneously in order to analyze the relationship between a driver’s driving performance and executive control functions and presented the results using performance efficiency and electroencephalogram (EEG) data [18]. This research presented evidence of the close relationship between executive control functions and driving performance [18]. The effect of painted sidewall patterns in tunnels—for their presence and their variability—on the brain activation of drivers has also been researched, comparing two groups of drivers: drivers with more than 1 year of driving experience and drivers with less than 1 year of driving experience [19]. Driving was not performed in person, and the research was conducted using recorded driving videos. The results presented that the activation of fusiform gyrus and precuneus were increased in tunnels with the decorated sidewall than that with an empty sidewall. Through prior research, it indicates that the presence of a decorated sidewall provides drivers with a better spatial and speed perception and could help reduce accidents associated with speed judgment [19]. The research team of this study observed effective connectivity of regions of the left and right hemispheres, as well as the entire brain, by extracting the brain regions activated during driving [12]. In the right hemisphere, the visual-attention pathway related to vision was discovered; in the left hemisphere, the inhibitory control movement and task-switching pathways related to cognitive processing of synesthesia were discovered. The visual attention, inhibitory control movement, and episodic memory retrieval pathways were evident in both hemispheres. It was observed through the pathways that driving requires multi-domain executive functioning and vision, and it is affected by the driver’s experience or familiarity. In the previous study, the research team investigated the effective connectivity between regions of the brain associated with driving. As described above, several studies on the differences in effective connectivity during driving and functional connectivity between regions of the brain during driving for specific subjects and specific cognition have been conducted.

However, no findings have been reported on how secondary tasks, which driving usually involves, affect the effective connectivity between the regions of the brain during driving. In other words, there has been no report on the types of connectivity in the left or right hemisphere and the entire brain when performing a secondary task during driving and their implications. Observing the neural activity of a specific region, as well as the effective connectivity between regions, will facilitate further investigation into the effect of secondary tasks on driving.

Therefore, this study aims to investigate the connectivity between the regions of the brain activated during driving and simultaneous secondary tasks using brain function images. To this end, dynamic causal modeling (DCM) is used to observe effective connectivity between regions in the left and right hemispheres and the entire brain.

## 2. Materials and Methods

### 2.1. Subjects

In this study, 15 adult males in their 20s (average 26.0 ± 1.4 years old) with no history of mental illness or neurological disease who had an average driving experience of 2.5 ± 1.6 years were selected as study subjects. It has been reported that gender differences for various driving performances (various road environments, avoidance behaviors, etc.) appear numerically different, but no statistical differences [20]. However, the male and female groups showed different levels of concern about risky situations or driving themselves while driving [21]. In other words, it was reported that men were less worried about unexpected situations while driving than women [21]. Based on the results of these previous studies, an experiment was conducted on men who are less afraid of the new driving environment. In addition, since this study focused on the results of effective connectivity during driving and distraction tasks, subjects were recruited by unifying gender. All the subjects were right-handed based on the results of the revised Edinburgh Reading Test [22]. Those with metallic substances in their body that may interfere with MR imaging, such as pacemakers and iron cores, or those with claustrophobia were excluded from recruitment. Before the experiment, external factors, such as smoking, alcohol, and coffee, which may affect driving performance and brain activation, were limited in the subjects, and the purpose and contents of the experiment were sufficiently explained. Driving was practiced until the subject became familiar with the driving simulator environment and was able to drive without any accident. The protocol for the research project has been approved by the Institutional Review Committee of Konkuk University, within which the work was undertaken, and it conforms to the provisions of the Declaration of Helsinki.

### 2.2. MR-Compatible Driving Simulator

**Figure 1:**
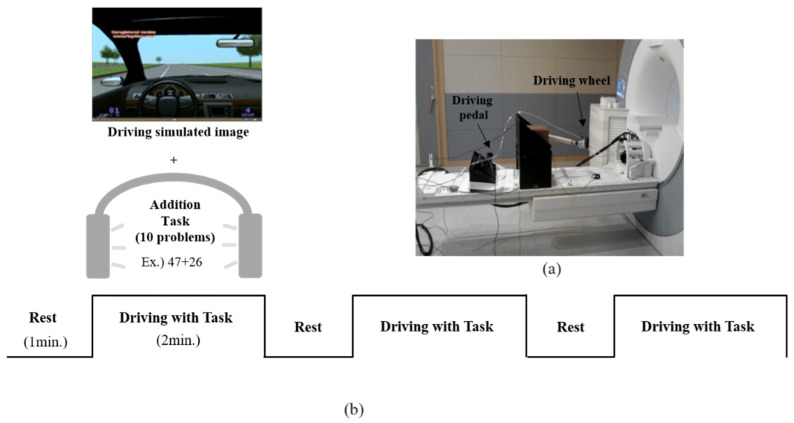
(**a**) MR-compatible driving simulator; (**b**) Experimental design, including driving environment and the secondary task (addition task).

This research team manufactured an MR-compatible driving simulator composed of a driving wheel and pedals (accelerator and brake), as shown in Figure 1a. The driving environment was presented using S/W by Lightrock Entertainment. The driving section was mostly straight, and a road with few factors that could distract the view of the subjects was selected (Figure 1b). The subjects operated the driving wheel with both hands, controlled the accelerator and brake with the right foot, and drove at a constant speed of 80 km/h without changing lanes. When operating the pedal with the right foot, the right pelvis was fixed, and the right foot of the subjects could comfortably touch the pedal with the right knee upright. In addition, the subjects were asked to operate the pedal using the right foot, ankle, and knee as much as possible. Visual information about driving was presented to the subject through a visual system mounted on the head coil. The configuration of the visual system was as follows: a resolution of 800 × 600 pixels; an aspect ratio of 4:3; field of view (FOV) of 30° horizontal/23° vertical.

### 2.3. Experimental Design

The experiment consisted of 3 blocks, and one block consisted of the Rest phase (1 min) and the Driving with Task phase (2 min), as shown in Figure 1b. During the rest phase, the subjects were asked to watch the still screen with their hands placed on the wheel and their right foot placed on the pedal without any movement. During the Driving with Task phase, the subjects were asked to simultaneously perform driving and a secondary task. The speed information of the driving vehicle was displayed on the lower left of the simulator screen so that the subject could maintain the specified speed of 80 km/h.

The additional task was performed as secondary. The additional tasks involving answering questions with the sum of the 2 digits not exceeding 100 after rounding up (ex. 32 + 42). A total of 30 questions, with 10 questions per block, were used for the additional tasks. The experimenter presented the task by voice through the audio system installed in the MR system, and the correct answer was confirmed after listening to the spoken answer of the subjects. The subjects were asked to focus on both the driving and the additional task. Oral driving cues were provided to the subjects during each driving phase.

### 2.4. Image Acquisition

The images were scanned with a 3T MRI system (Magnetom TrioTim, Siemens Medical Systems, Erlangen, Germany) using a standard 32-channel head coil. Single-shot echo planar fMRI scans were acquired in 29 continuous slices, parallel to the anterior commissure-posterior commissure line. The parameters for fMRI were: TR/TE = 3000/30 ms, FOV = 200 mm, flip angle = 90°, matrix = 128 × 128, slice thickness = 5 mm, voxel size = 1.6 × 1.6 × 5.0 mm. Anatomical images were obtained using a T1-weighted 3D MPRAGE sequence with the following parameters: TR/TE = 1900/2.48 ms, FOV = 200 mm, flip angle = 9°, matrix = 256 × 256, slice thickness = 1 mm, voxel size = 0.8 mm × 0.8 mm × 1.0 mm.

### 2.5. Image Analysis

The fMRI data were analyzed with SPM 12 software (Wellcome Department of Cognitive Neurology, London, UK). All functional images were aligned with the anatomic images of the study using affine transformation routines built into the SPM 12 program. Realign a time-series of images acquired from the same subject using a least-squares approach and a 6 parameter (rigid body) spatial transformation. The first image in the list specified by the user is used as a reference to which all subsequent scans are realigned. The realigned scans were coregistered with the participant’s anatomic images obtained during each session and normalized to a template image in SPM 12, which uses the space defined by the Montreal Neurologic Institute. The motion correction was performed using a Sinc interpolation. Time-series data were filtered with a 240-s high-pass filter to remove artifacts due to cardiorespiratory and other cyclical influences. Additionally, the coregistered T1 and T2 images were used in a multichannel segmentation routine to extract probabilistic maps of 6 tissue classes: GM, WM, CSF, bone, soft tissue, and residual noise. The functional map was smoothened with an 8-mm isotropic Gaussian kernel before the statistical analysis. The statistical analysis was performed at the group level using the general linear model and the theory of Gaussian random fields implemented in SPM 12. Group analysis was performed to extend the inference of the individual activation to the general population from which the subjects were drawn to list all clusters above the chosen level of significance, as well as the separate (>8 mm apart) maxima within each cluster, with the details of significance thresholds and search volume underneath.

The subtraction method was used to extract the active regions associated with driving during the Driving with Task phase compared with the Rest phase (Driving with Task phase—Rest phase).

### 2.6. Connectivity Analysis

To extract the effective connectivity between the active regions, the causal relationship between the regions of interest was analyzed using dynamic causal modeling (DCM). DCM is a model-based analysis method that is not only applied to brain activation analysis by the general linear model (GLM) but also the analysis of the connectivity between the regions of the brain. It estimates the relationships between the variables using analysis of covariance or linear regression analysis and builds a correlation model between domains. DCM is performed by variable estimation and Bayesian model selection (BMS) after defining a model in MATLAB-based statistical parametric mapping (SPM). DCM analyzes the correlation between regions using the blood-oxygen-level-dependent (BOLD) signals of each region and establishes an optimal dynamic causal model under the assumption that all active regions form a network [23].

**Figure 2:**
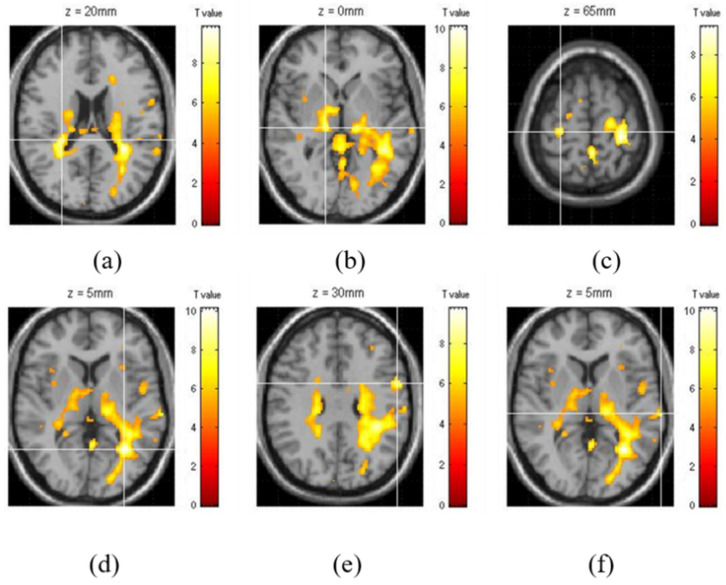
The 3 regions in the right hemisphere with the highest z-scores for the secondary task while driving (white crossbar: each region): the (**a**) insula (Ins), (**b**) thalamus (Th), and (**c**) precentral gyrus (PrG). The 3 regions in the left hemisphere: the (**d**) lingual gyrus (LiG), (**e**) precentral gyrus (PrG), and (**f**) superior temporal gyrus (STG).

Among the activated regions, 3 regions of the right hemisphere and 3 regions of the left hemisphere with the highest z-scores were selected and analyzed for their effective connectivity. The 3 regions of the right hemisphere with the highest z-scores while simultaneously driving and performing a secondary task were selected; they were the insula (Ins), thalamus (Th), and precentral gyrus (PrG). The 3 regions of the left hemisphere that were selected were the lingual gyrus (LiG), precentral gyrus (PrG), and the superior temporal gyrus (STG) (Figure 2). The time-series BOLD signal of the region of interest was extracted from the sphere region with a diameter of 5 mm.

The effective connectivity between the 3 regions in each of the right and left hemispheres and the 6 regions of the entire brain were analyzed. The effective connectivity analysis was divided into (1) selecting the driving input region for the region of interest and (2) establishing a model by analyzing the connectivity between the regions of interest using the correlation of the BOLD signal.

First, an analysis was performed to designate driving inputs of the right and left hemispheres (3 regions) and the entire brain (6 regions). After designating the regions of interest as fully connected (all regions of interest are connected), a model was established that assumed each region as an input. For example, after designating 3 regions of the right hemisphere as fully connected, 3 models were established, with each using the insula (Ins), thalamus (Th), and precentral gyrus (PrG) as driving input. The most meaningful model was determined through the calculation of the fixed effect using BMS.

**Figure 3:**
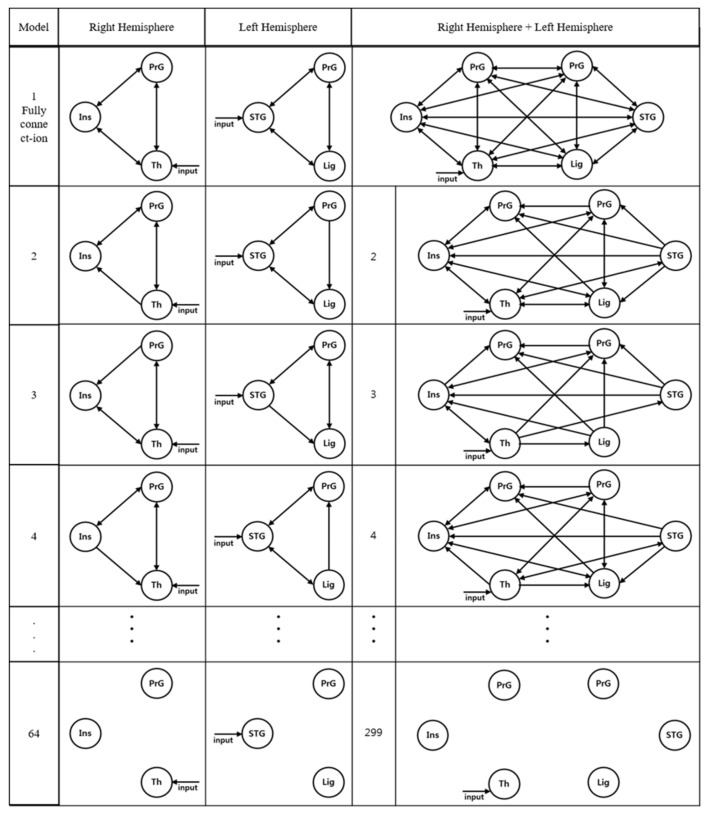
The model for estimating the effective connectivity of the regions of interest while performing the secondary task and driving (3 regions in each of the left and right hemispheres and 6 regions in the entire brain).

Second, after designating the driving input regions of the right and left hemispheres and the entire brain, the connectivity between the regions of interest was analyzed. Sixty-four models were created for each of the right and left hemispheres (Figure 3). The first and second columns of Figure 3 show the models for the 3 regions of the right and left hemispheres, respectively, and the third column shows the model for the 6 regions of the entire brain. As shown in the first and second columns, model 1 was a fully connected model representing an intrinsic connection, and all regions were connected in both directions. For models 2–63, the direction of connection was changed for each region based on the external connection. Model 64 was set as a model without an inter-regional connection.

The connectivity of the 6 regions of the entire brain was represented by 299 models. The results are shown in the third column of Figure 3. Similar to the analysis method above, model 1 was configured as a fully connected model, models 2–298 were configured as the predicted models with changes in the connectivity between the regions of interest, and model 299 was configured as a model without an inter-regional connection.

This analysis was performed for each subject; the posterior probability for each model was extracted for each subject based on a comparison of the models generated for each hemisphere using the fixed effects (FFX) of BMS. The random fixed effect (RFX), which is provided in BMS, was used to compare the models in the group analysis based on subject-specific data. The RFX is used to obtain the optimal probability values for the hypothesized models, and it has been used to estimate the probability values for models. By verifying this at the group level, the effective connectivity was determined by estimating the average value of the correlation between the regions for the model with the highest probability value.

## 3. Results

The average driving speed of the subjects was 73.5 ± 6.7 km/h, and the correct answer rate was 78.5 ± 11.7%, indicating that the driving and the additional tasks were properly performed.

**Figure 4:**
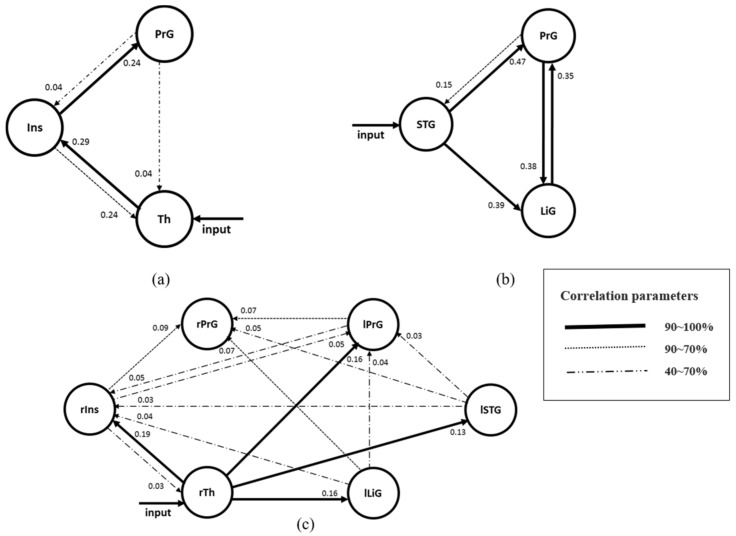
The model for the effective connectivity of the regions in the (**a**) right hemisphere, (**b**) left hemisphere, and (**c**) the entire brain while performing a secondary task and driving.

When driving and distraction tasks are performed simultaneously, the three regions of the right hemisphere with the highest z-scores were the insula (Ins), thalamus (Th), and precentral gyrus (PrG) (Figure 2a–c; their z-scores were 8.65, 8.25, and 8.87, respectively. After calculating the fixed effect for the three models with each of the three regions as the driving input, the most significant model was the model with the thalamus as the driving input (Probabilities: 1.00, C-direct effects: 0.64 Hz). The connectivity between the three regions of the right hemisphere showed the greatest connectivity from the thalamus to the insula (A-intrinsic connections: 0.29, correlation parameters: 100%) and from the insula to the precentral gyrus (0.24, 96%) (Table 1, Figure 4a). The connectivity from the insula to the thalamus also demonstrated a high correlation of 90–70% (Table 1, Figure 4a).

In the left hemisphere, the three regions with the highest z-scores were the lingual gyrus (LiG), the precentral gyrus (PrG), and the superior temporal gyrus (STG), as shown in Figure 2 d–f. The z-scores were 6.33, 8.87, and 6.63, respectively. After calculating the fixed effect for the three models with each of the three regions as the driving input, the most significant model was the model with the superior temporal gyrus as the driving input (Probabilities: 1.00, C-direct effects: 0.23 Hz). With the exception of the connectivity from the lingual gyrus to the superior temporal gyrus, all the other connectivities were high (Table 1, Figure 4b).

For the entire brain, the effective connectivity of the right insula (rIns), the right thalamus (rTh), and the right precentral gyrus (rPrG) of the right hemisphere and the left lingual gyrus (lLiG), the left precentral gyrus (lPrG), and the left superior temporal gyrus (lSTG) of the left hemisphere were analyzed (Table 2, Figure 4c). Among the models using the six regions as driving inputs, the most meaningful model was the model with the right thalamus as the driving input (Probabilities: 1.00, C-direct effects: 0.83 Hz). The effective connectivity from the right thalamus to the right insula (A-intrinsic connections: 0.19, correlation parameters: 100%), left precentral gyrus (0.16, 99%), left superior temporal gyrus (0.13, 99%), and left lingual gyrus (0.16, 100%) was high.

## 4. Discussion

In this study, the connectivity of the regions of the right and left hemispheres and the entire brain associated with various cognitive processes required during driving and simultaneous secondary tasks was analyzed. The effective connectivity between the active regions of each hemisphere and the entire brain was used to represent the input region related to the connectivity and the direction of the connectivities from the input region to other regions and their correlation, as well as analyze the implications of each connectivity.

### 4.1. Effective Connectivity between Regions Activated in the Right Hemisphere

In the right hemisphere, the thalamic region serving as a relay between the motor regions was mainly designated as the driving input. The thalamus, as a subcortical motor center, has been reported to play an essential role in neuronal information processes for motor control [24,25]. The thalamic regions were also involved in the generation of antisaccade eye movement (that is, the ability to inhibit the reflexive jerking movement of the eyes in the direction of a presented stimulus) [25]. The insula is involved in motor control, especially hand-and-eye motor movement and motor control, and it showed high connectivity to the primary motor cortex and precentral gyrus [26,27]. The motor pathway from the thalamus to the motor cortex (precentral gyrus) is well-known based on previous reports [28], and this study found that the insula was involved in the connection between the thalamus and the PrG in the right hemisphere during driving and simultaneous secondary tasks. The insula is known to play various roles in perception, motor control, cognitive functioning, interoceptive awareness, and cognitive control [29,30]. Based on the results of these previous studies, new connectivities involving the insula in the motor pathway (thalamus–precentral gyrus) can be inferred as follows.

During the movement caused by the task, the connectivity from the thalamus to the insula seemed high for controlling the unnecessary movements during driving and the simultaneous secondary tasks. The connection was determined to be large, as the control of movement related to driving performance was transmitted to the precentral gyrus region. In other words, a direct motor pathway from the thalamus to the precentral gyrus region has been proposed in general [25], but the insula was involved in the connection between these two regions due to the secondary task presented in this study. The connectivity to this region enabled the control of the unnecessary movements (motor control) caused by the secondary task. Therefore, the effective connectivity (thalamus to insula to precentral gyrus) of the three regions activated in the right hemisphere is to be defined as the motor control pathway.

### 4.2. Effective Connectivity between Regions Activated in the Left Hemisphere

In the left brain, the superior temporal gyrus was designated as the driving input region. The superior temporal gyrus has been reported as the region mainly involved in auditory perception and responsible for understanding language [31]. The left superior temporal gyrus was reported as an active region during the performance of the additional task [32,33]. In this study, the experimenter verbally provided the subjects with driving instructions during each phase (“Please drive” and “Stop driving”), and the questions for the additional task, which was the secondary task, were presented by voice; therefore, the superior temporal gyrus seemed to be designated as the input region.

As mentioned in Section 4.1, it is well-known that the precentral gyrus region is the primary motor cortex region responsible for controlling the voluntary motor movement of the body’s contralateral side [34].

Of the three regions of the left hemisphere, the lingual gyrus is the first extrastriate visual processing region activated when performing tasks, including visual and motor imagery [35,36]. Second, it was reported that the lingual gyrus was modulated even when performing tasks related to spatial attention [37]. Third, some studies have reported that the lingual gyrus is activated during limb movements [38]. Fourth, it has been reported that the lingual gyrus is a region that becomes active during tasks such as declarative memory [39,40]. Declarative memory is a memory that consciously recalls the knowledge gained through learning. Descriptive memory can be broadly classified into episodic memory, which is influenced by the subjective experience of a specific individual, and semantic memory, which stores only objective facts. Declarative memory is different from procedural memory, which is stored unconsciously by repetition and can be used immediately when necessary. In other words, it processes visual language information and plays an important role in the analysis of encoded visual memories [41].

As described above, the connectivities from the precentral gyrus to the lingual gyrus and from the lingual gyrus to the precentral gyrus were demonstrated, starting from the superior temporal gyrus, due to the secondary task (presented as a specific task, addition task) based on the functions of the three regions activated in the left hemisphere. The precentral gyrus, which is involved in the voluntary movement of steering wheel manipulation with both hands and pedal manipulation using the right foot (ankle and knee), performs the functions of limb movement, visual processing, and spatial attention. The connectivity to the lingual gyrus, which processes information for visual recognition and grasping in the driving environment, appeared to be dominant. Based on the memory of the subjects about the previous driving experience, recalling declarative memory, focusing on the driving environment, and moving for the manipulation of the wheel and pedals while driving should have been demonstrated. In other words, the connectivity between the regions related to the secondary task and the movements (both hands, right feet, ankles, and knees) needed during driving based on the driving experience (memory) of the subjects was dominant. Based on these functions, the connectivity of the three regions of the left hemisphere can be considered as the motor network of declarative memory (also known as the visual-motor decision pathway or motor skill pathway).

The superior temporal gyrus, the input region of the left brain, is thought to be a characteristic region based on the design (addition task, voice presentation) of this study, and the involvement of this region may differ with the type and method of the presented secondary task. In the left brain, bidirectional connectivity between the precentral and lingual gyri appears to have important implications.

### 4.3. Effective Connectivity between Regions Activated in Both Hemispheres

Of the six active regions of the entire brain, the right thalamus has been designated as the input region, as it plays an essential role in neuronal information processes for motor control as a subcortical motor center. It also has sensorimotor functions, as mentioned above. It was somewhat expected that the right thalamus was selected as the input region out of the six regions for driving, which involves extensive touching and movements, including holding the steering wheel with both hands and pedaling with the right foot. Starting from the right thalamus, which was the input region, the outcomes of the analysis of all connectivities with a correlation of 70% or higher (Figure 4c) are as follows:

① The connectivity of the right thalamus, right insula, and right precentral gyrus is the motor control pathway within the right hemisphere. The integrated analysis of the left and right hemispheres confirmed that the connectivity of the three regions of the right hemisphere was dominant.

② The connectivity of the right thalamus, left precentral gyrus, and right precentral gyrus was also a pathway related to the voluntary movement for driving, and only the connectivity from the left precentral gyrus to the right precentral gyrus. The precentral gyrus connectivity in both hemispheres was expected, but no connectivity from the right precentral gyrus to the left precentral gyrus was observed. The connectivity of rPrG appeared more dominant in lPrG because the steering wheel was operated with both hands after operating the pedal with the right limb during driving.

③ Two facts were inferred from the connectivity of the right thalamus, left lingual gyrus, and right precentral gyrus. First, it has been reported that declarative memory, including episodic memory and semantic memory, is predominantly activated in the left lingual gyrus [39,42]. During driving, movement is affected by the memory of the subject’s driving experience. Therefore, this connectivity is considered as a more explicit pathway of the motor network of declarative memory (also known as the visual-motor decision pathway or motor skill pathway) mentioned in Section 4.2. Second, as in the connectivity of ② above, the connectivity underlying the movement of both hands after the movement of the pedal with the right foot (right-limb movement) was dominant.

④ The connectivity of the right thalamus and left superior temporal gyrus seems to have emerged in relation to the secondary task. Although the correlations of the domains were low, the connectivity of the left superior temporal and right insula was observed. The connectivity from the left superior temporal gyrus to the right precentral gyrus and left precentral gyrus was also observed. Although the attention-distribution task was performed during driving, it was confirmed that the connection between the region related to the distraction task and the driving performance did not show a high correlation. In other words, the region associated with the secondary task was activated, but the task did not appear to substantially affect the driving performance.

## 5. Conclusions

This study examined the effective connectivity of the left and right hemispheres and the entire brain. When simultaneously performing driving and secondary tasks, a motor control pathway related to movement control for driving performance was observed in the right hemisphere. The motor network of declarative memory (also known as visual-motor decision pathway or motor skill pathway) was also observed in the region related to movement control for driving performance, starting with the region in the left hemisphere related to the additional task, which was the secondary task. In the six regions of the entire brain, the pathways in each of the right and left hemispheres were observed.

The motor network of declarative memory (also known as visual-motor decision pathway or motor skill pathway), which is the connectivity of the right thalamus, left lingual gyrus, and right precentral gyrus, was interesting. Although it was limited to the task of driving, the results seem meaningful as they show the connectivity underlying voluntary movement related to memory from human experience. The present study suggests connectomic features of the left and right hemispheres in different driving states. Such results can be significant as a feature for distinguishing different driving statuses. While the addition task used in the current research was the “secondary task while driving,” it can be hypothesized that connectomic features would be different for various secondary tasks, such as talking while driving, manipulating a mobile phone/GPS navigation, and food consumption. Results of the current study are just parts of multivariate data that could be derived from performing various secondary tasks while driving and are thought to be significant as foundational data for distinguishing driving statuses. This study is also considered to be of great significance; it is the first to generally and comprehensively investigate the effective connectivity between regions of the brain associated with the performance of a secondary task while driving.

Since the secondary task in this study was not distracting enough during the actual driving situation, the connectivity between the regions associated with the secondary task and the regions associated with the driving performance was not large. Therefore, further studies on the effect of distraction during driving and effective connectivity are necessary, and a more realistic secondary task should be used. Moreover, comparisons should be made according to gender (male, female), age (20s~70s), subgroups (Control vs Post Traumatic Stress Disorder, etc.), various brain imaging systems (Electroencephalography, EEG/Diffusion Tensor Imaging, DTI/B-spatial distribution in DTI [43,44], BSD-DTI [43,44], and so on), and driving experience (novice drivers, experienced drivers) since the subjects of this study only consisted of men in their twenties with limited driving experience. Dynamic causal modeling, utilized in the analysis section of the present study, has the following limitations. It is not ideally suited for exploratory analyses. Although methods have been implemented for automatically searching over reduced models (Bayesian Model Reduction) and for modelling large-scale brain networks, these methods require an explicit specification of model space. Therefore, in the research team’s future endeavors, the newest brain connectome analysis techniques (e.g., resting-state functional connectivity) should be applied for deriving various results, such as individual patterns and multi-view features.

## Figures and Tables

**Table 1:** Correlations of the three regions in each of the right and left hemispheres while performing a secondary task while driving.

			**From**		
			**Ins**	**Th**	**PrG**
Right Hemisphere	To	Ins		0.29 (100%)	0.04 (57%)
		Th	0.24 (83%)		0.04 (57%)
		PrG	0.24 (96%)	0	
			**From**		
			**LiG**	**PrG**	**STG**
Left Hemisphere	To	LiG		0.38 (96%)	0.39 (98%)
		PrG	0.35 (94%)		0.47 (99%)
		STG	0	0.15 (74%)	

Insula (Ins), Thalamus (Th), Precentral Gyrus (PrG), Lingual Gyrus (LiG), Precentral Gyrus (PrG), and Superior Temporal Gyrus (STG).

**Table 2:** Correlations of the six regions in the entire brain while performing a secondary task while driving.

		From					
		rIns	rTh	rPrG	lLiG	lPrG	lSTG
To	rIns		0.19 (100%)	0	0.04 (62%)	0.05 (64%)	0.03 (60%)
	rTh	0.03 (58%)		0	0	0	0
	rPrG	0.09 (76%)	0		0.07 (70%)	0.07 (72%)	0.05 (58%)
	lLiG	0	0.16 (100%)	0		0	0
	lPrG	0.05 (64%)	0.16 (99%)	0	0.04 (60%)		0.03 (65%)
	lSTG	0	0.13 (99%)	0	0	0	

r: right hemisphere/l: left hemisphere.

## Data Availability

The datasets generated for this study are available on request to the corresponding author.
